# Thermogravimetric and kinetic analysis to discern synergy during the co-pyrolysis of microalgae and swine manure digestate

**DOI:** 10.1186/s13068-019-1488-6

**Published:** 2019-06-29

**Authors:** Arun K. Vuppaladadiyam, Hao Liu, Ming Zhao, Abdul F. Soomro, Muhammad Zaki Memon, Valerie Dupont

**Affiliations:** 1 School of Environment, Tsinghua University, Beijing, 100084 China; 2 Beijing Guohuan Tsinghua Environmental Engineering Design & Research Institute Co., Ltd., Beijing, China; 3 School of Chemical and Process Engineering, The University of Leeds, Leeds, LS2 9JT UK

**Keywords:** Microalgae, Manure, Co-pyrolysis, Isoconversional, Kinetics, Thermogravimetric

## Abstract

**Background:**

Co-pyrolysis of wastes with other feedstock can synergistically improve the rate of biomass decomposition and also help to resolve the issues related to limited availability feedstock. In this regards, synergistic interaction between feedstock during co-pyrolysis is an important aspect of research. As the constituents of aquatic and lignocellulosic biomass are different, and the decomposition pattern of aquatic biomass is dissimilar when compared to lignocellulosic biomass, it is important to understand whether these two biomasses interact during co-pyrolysis.

**Results:**

Synergism in the co-pyrolysis of microalgae (MA), swine manure digestate (SWD), and their blends (MA/SWD) (w/w %), 2.5/7.5 (MD-1), 5/5 (MD-2), and 7.5/2.5 (MD-3), was evaluated based on decomposition behavior, gas yields, extent of thermal degradation, and kinetics. Extractives and volatiles in biomass enhanced the reaction kinetics and products yields, as indicated by the reduction in apparent activation energy of the blends, accompanied by an increase in H_2_, total gas yield, and extent in degradation. Thermogravimetric data, via isoconversional methods, were interpreted to achieve the apparent activation energies for the thermal degradation of the MA, SWD, and their blends. The best fit reaction models were identified using compensation effect and generalized master plots methods. Semi-quantitative method was used to quantify the evolved gas species. H_2_, CO, and CO_2_ were noted to be the dominant gases, implying that tar cracking and reforming reactions were predominant.

**Conclusions:**

Overall, synergy was noticed with respect to the pyrolysis of SWD biomass to gas products in the presence of MA biomass, whereas synergy was witnessed up to 50 w/w % MA in view of kinetic parameters as evaluation criteria.

## Background

The alternative energy sources, particularly biomass derived biofuels, have been highlighted as substitutes for conventional petroleum fuels. Though, there are numerous advantages associated with biofuels, the first-generation biofuels (produced from edible crops) could result in diverse side effects such as water shortage, increase in the price of edible crops, etc. The negative effects associated with the first-generation biofuels, to a major extent, can be eliminated by shifting to the second generation (produced form non-edible biomass) or third generation (produced from aquatic biomass such as macro-/microalgae) as they do not present any threat to food chain or ecosystem [1]. Moreover, microalgae can grow in fresh water, brackish, and wastewater, making them suitable for simultaneous wastewater treatment and biomass generation [2]. In addition, microalgae cultivation can be integrated with large CO_2_ point sources, such as power or cement plants, and wastewater treatment facilities, making it a sustainable pathway for biomass generation coupled with carbon capture [3].

The livestock sector has been reported to be one of the principal contributors to grave environmental issues around the world. In China, it is reported that animal manure is one of the major sources of water pollution, mainly through the transfer of nitrogen (N) and phosphorus (P) [4]. Furthermore, it is estimated that 837 million tons of animal manure are produced in China, 208 million of which contributed by swine manure alone [5]. These considerable amounts of wastes present a serious threat to the environment if they are not managed properly. Furthermore, swine manure is more recognized as a pollutant and is often a derelict bioenergy resource. However, use of manure as a bioenergy feedstock could reduce waste disposal problems and alleviate pressure on environment by providing clean energy [6]. In particular, generation of energy from livestock manure eliminates the most common problems, such as unwanted transfer of pathogens into ecosystem, eutrophication caused by the leaching of nutrients into nearby water bodies, etc., associated with the conventional means of waste management [7].

A wide range of technologies are available for converting biomass to bioenergy, which includes biochemical such as anaerobic digestion, transesterification, fermentation, etc., and thermochemical techniques, such as gasification, liquefaction, pyrolysis, etc. [2]. Till date, anaerobic digestion (AD) has been widely acknowledged as a means to add value and stabilize solid wastes. However, the high CO_2_ content of biogas generated from AD process impairs the fuel quality and necessitates several purification steps. In addition, the carbon deposited in the microorganisms lowers the carbon conversion from manure to biofuels. Among the thermochemical conversion techniques, pyrolysis has been the most widely accepted technology to convert biomass, especially agricultural and aquatic biomass, into bioenergy owing to its potential advantages such as less pollution emission, reasonable cost, and simple operation [8]. However, co-pyrolysis and catalytic pyrolysis help to improve the nature, quantity, and quality of the end products [9]. Furthermore, it is reported that co-pyrolysis of wastes with other feedstock can synergistically improve the rate of biomass decomposition and also help to resolve the issues related to limited availability feedstock [10]. In this regards, synergistic interaction between feedstock during co-pyrolysis is an important aspect of research. As the constituents of aquatic and lignocellulosic biomass are different, and the decomposition pattern of aquatic biomass is dissimilar when compared to lignocellulosic biomass, it is important to understand whether these two biomasses interact during co-pyrolysis. In the former, because of its nutritional value, microalgae decomposition has been mainly related to its lipids, proteins, and carbohydrates content [11], while the latter, intended as biofuel, the decomposition has been mainly studied in terms of its cellulose, hemicellulose, and lignin content [12].

The knowledge of pyrolysis kinetics is essential for many reasons such as predicting the behavior of biomass during pyrolysis which forms the basis for reactor design. A number of successive and/or parallel reactions occur during the thermal breakdown of biomass making it a complicated process. For a thermal decomposition process, the International Confederation for Thermal Analysis and Calorimetry (ICTAC) highly recommends *iso*conversional methods to identify the ‘kinetic triplet’ that include apparent activation energy, pre-exponential factor, and reaction mechanism [13, 14]. In the present study, an attempt has been made to evaluate the synergistic influence of microalgae and swine manure digestate during co-pyrolysis as compared to individual pyrolysis. Thermal decomposition behavior of two different category of biomass, namely microalgae and swine manure digestate, and their blends in different ratios have been studied using thermogravimetric analyser coupled with mass spectrometer. Furthermore, the kinetic parameters, which include apparent activation energy (*E*_*α*_), and pre-exponential or frequency factor (*A*) were identified using isoconversional methods (Kissinger–Akahira–Sunose (KAS) [15] and Flynn–Wall–Ozawa (FWO) [16, 17] methods) and compensation effect respectively. The reaction model, *f*(*α*), was identified using compensation effect [18] and generalized master plots method [19]. Furthermore, the gases evolved during pyrolysis were analyzed and reported. The most commonly used reaction mechanisms, along with their differential *f*(*α*) and integral expressions *g*(*α*), are presented in Table 1.

**Table 1:** Common solid-state reaction mechanisms [44, 45]

Reaction mechanisms	Differential form *f*(*α*)	Integral form *g*(*α*)
A_2_—Nucleation and nuclei growth (Avrami Eq. 1)	$2(1 - \alpha )[ - \ln (1 - \alpha )]^{1/2}$	$[ - \ln (1 - \alpha )]^{1/2}$
A_3_—Nucleation and nuclei growth (Avrami Eq. 2)	$3(1 - \alpha )[ - \ln (1 - \alpha )]^{3/2}$	$[ - \ln (1 - \alpha )]^{1/3}$
A_4_—Nucleation and nuclei growth (Avrami Eq. 3)	$4(1 - \alpha )[ - \ln (1 - \alpha )]^{3/4}$	$[ - \ln (1 - \alpha )]^{1/4}$
R_2_—Phase boundary controlled reaction (contracting area)	$2(1 - \alpha )^{1/2}$	$[1 - (1 - \alpha )]^{1/2}$
R_3_—Phase boundary controlled reaction (contracting volume)	$3(1 - \alpha )^{2/3}$	$[1 - (1 - \alpha )]^{1/3}$
D_1_—One-dimensional diffusion	$(1/2)\alpha$	$\alpha^{2}$
D_2_—Two-dimensional diffusion (Valensi equation)	$[ - \ln (1 - \alpha )]^{ - 1}$	$(1 - \alpha )\ln (1 - \alpha ) + \alpha$
D_3_—Three-dimensional diffusion (Jander equation)	$(3/2)[1 - (1 - \alpha )^{1/3} ]^{ - 1} (1 - \alpha )^{2/3}$	$[1 - (1 - \alpha )^{1/3} ]^{2}$
D_4_—Three-dimensional diffusion (Ginstling–Brounshtein equation)	$(3/2)[1 - (1 - \alpha )^{1/3} ]^{ - 1}$	$[1 - (2/3)\alpha )] - (1 - \alpha )^{2/3}$
F_1_—Random nucleation with one nucleus on the individual particle	$1 - \alpha$	$- \ln (1 - \alpha )$
F_2_—Random nucleation with two nuclei on the individual particle	$(1 - \alpha )^{2}$	$1/(1 - \alpha )$
F_3_—Random nucleation with three nuclei on the individual particle	$(1/2)(1 - \alpha )^{3}$	$1/(1 - \alpha )^{2}$
P_1_—Mampel power law $$ \left( {n \, = \,{\raise0.7ex\hbox{$1$} \!\mathord{\left/ {\vphantom {1 2}}\right.\kern-0pt} \!\lower0.7ex\hbox{$2$}}} \right) $$	$$ 2\alpha^{1/2} $$	$$ \alpha^{1/2} $$
P_2_—Mampel power law $$ \left( {n \, = \,{\raise0.7ex\hbox{$1$} \!\mathord{\left/ {\vphantom {1 3}}\right.\kern-0pt} \!\lower0.7ex\hbox{$3$}}} \right) $$	$$ 3\alpha^{2/3} $$	$$ \alpha^{1/3} $$
P_3_—Mampel power law $$ \left( {n \, = \,{\raise0.7ex\hbox{$1$} \!\mathord{\left/ {\vphantom {1 4}}\right.\kern-0pt} \!\lower0.7ex\hbox{$4$}}} \right) $$	$$ 4\alpha^{3/4} $$	$$ \alpha^{1/4} $$

## Methods

### Biomass preparation and characterization

The microalgae, *Spirulina platensis* (MA), sample was collected from Phycospectrum Environmental Research Centre (PERC), Chennai, Tamil Nadu, India. The isolated culture was then inoculated and grown in a 1 L Erlenmeyer flask with working volume of 500 mL using CFTRI (developed by Central Food and Technology Research Institute, Mysore, India) [20] medium for 30 days. The composition of CFTRI media is presented in Table 2. The cultivation was carried out in an incubator, maintaining a temperature of 30 °C and a light intensity of 500 lx throughout the cultivation period. After the cultivation phase, the microalgae cultures were harvested by phase separation in a centrifuge at 6500 rpm for 15 min to obtain the microalgae biomass. The solid phase biomass was washed with deionized water multiple times and was maintained at 80 °C in a ventilated oven to procure dried algal biomass.

**Table 2:** Composition of CFTRI media [46, 47]

Chemicals	g/L dH_2_O
NaHCO_3_	4.5
K_2_HPO_4_	0.5
NaNO_3_	1.5
K_2_SO_4_	1
NaCl (Crude)	1
MgSO_4_·7H_2_O	0.2
CaCl_2_	0.04
FeSO_4_	0.01

Partially digested manure was collected from swine manure anaerobic digestion plant Donghua, Beijing. The Automatic Methane Potential Test System II (Bioprocess Control, Sweden) was used to completely digest the sample. The digestion process was run in triplicates, in a 0.6 L reactors, to ensure the complete digestion. The system consisted of three units: Unit A is a water bath containing 15 glass bottles for anaerobic digestion (AD) and is maintained at mesophilic temperature (35 °C); Unit B, CO_2_ adsorption using 3 M sodium hydroxide (NaOH); and Unit C, in which the volume of CH_4_ released from Unit A was automatically recorded. A mixing rod with slow mechanical rotation was used in each bottle in Unit A. The glass bottles containing manure samples were placed in the water bath in Unit A and only gas generated during the digestion process is allowed to pass to Unit B, where bromothymol blue indicator is used to monitor the change in the pH. However, there is no possibility of the manure samples in Unit A to react with 3 M NaOH in Unit B. The run was stopped after ensuring the methane generation was less than 10 mL per day. The growth and digestion characteristics of MA and swine manure digestate (SWD), respectively, as they are out of the scope of current study, are not discussed. Elemental analysis was undertaken using a carbon–hydrogen–nitrogen analyzer (model CE 440; EAI, Oakland, NJ, USA), biocompounds (lipid, protein, and carbohydrate) analysis [21], and structural component analysis (lignin, cellulose, and hemicellulose) [22] were carried out as per standard procedures and are presented in Table 3.

**Table 3:** Proximate analysis, elemental composition, and chemical composition of biomass samples

Parameters	Sample	MA	SWD
Proximate analysis (wt%)	Moisture	4.76 ± 0.238	3.9 ± 0.195
	VM	84.25 ± 2.527	73.76 ± 2.950
	FC	5.85 ± 0.293	12.62 ± 0.631
	Ash	5.14 ± 0.103	9.72 ± 0.243
Elemental composition (wt%)	C	47 ± 1.88	42.3 ± 1.904
	H	6.8 ± 0.34	6.1 ± 0.305
	O	27.8 ± 1.39	41.3 ± 2.065
	N	10.5 ± 0.53	1.4 ± 0.07
	S	0.82 ± 0.041	0.71 ± 0.036
Chemical composition (wt%)	Carbohydrate	19.8 ± 0.99	21.3 ± 1.065
	Protein	65.2 ± 1.956	16.3 ± 0.408
	Lipid	8.7 ± 0.131	7.8 ± 0.156
Structural component analysis	Lignin		6.49 ± 0.149
	Cellulose		59.31 ± 2.966
	Hemicellulose		14.7 ± 0.441

### TG–DTG–MS analysis

Thermal analyser (Q600) was used to perform the thermogravimetric (TG) and derivative thermogravimetric (DTG) analyses of individual and blended biomasses. Sample, ca. 3 mg weight, was heated to 800 °C from ambient temperature. Initially, a constant heating rate of 15 °C min^−1^ was used, and later, the experiments were run at different heating rates of 10 and 20 °C min^−1^, from room temperature to 800 °C. For all the experiments, argon (Ar) gas was used as a purge gas and the flow was set at 500 mL min^−1^ to ensure an inert environment. The results reported are the average of the data obtained by conducting the experiments in triplicate.

The pyrolysis gas was collected and delivered to the mass spectrometer (MS) by heated capillary, wherein the gas molecules are ionized and differentiated based on their mass-to-charge ratio (*m/z*). The ions that were scanned and their respective evolved gases are presented in Table 4.

**Table 4:** Ion fragments and their representative gas species

*m/z*	Ion fragments	Representative species
2	H_2_^+^	Hydrogen
15	CH_4_^+^	Methane
28	CO^+^	Carbon monoxide
40	Ar^+^	Argon
44	CO_2_^+^	Carbon dioxide

With respect to the purge flow rate of Ar and weight of biomass, normalization of the raw signals from MS was done based on the following equation:

1 $$ {\text{Normalized signal for key molecule fragments }}`i '= \text{ }{{\left( {{\text{IC}}_{i} *500} \right)} \mathord{\left/ {\vphantom {{\left( {{\text{IC}}_{i} *500} \right)} {\left( {{\text{IC}}_{\text{Ar}} *{\text{wt}}_{\text{sample}} } \right)}}} \right. \kern-0pt} {\left( {{\text{IC}}_{\text{Ar}} *{\text{wt}}_{\text{sample}} } \right)}}, $$

where, IC_*i*_ and IC_Ar_ indicate molecular *m/z* signals for molecular ion fragments and Ar, ‘*i*’ (arbitrary unit), and wt_sample_ indicate the weight of biomass sample (*g*). The detailed procedure for the analysis of evolved gas species is discussed in our previous study [23].

### Kinetic analyses

Biomass pyrolysis varies for different biomass, mainly because of differences in their chemical structure. However, the overall path of biomass pyrolysis can be defined as follows: ${\text{Biomass}}\, \to {\text{Char}}\, + \,{\text{Volatiles}}\, + \,{\text{Gases}}.$ The rate constant *k*(*T*), according to the Arrhenius equation, can be expressed as follows:

2 $$ k(T) = {\text{Ae}}^{{\left( {\frac{{ - E_{\upalpha} }}{RT}} \right)}} , $$

where *A* (s^−1^) and *E*_*α*_ (J mol^−1^) are pre-exponential and apparent activation energy of the reaction, respectively, and *R* and *T* (°K) are universal gas constant (8.314 J mol^−1^ K^−1^) and absolute temperature, respectively. The kinetics of solid-state thermal degradation can be defined as follows:

3 $$ \frac{{{\text{d}}\alpha }}{{{\text{d}}t}} = k(T)f(\alpha ) = {\text{Ae}}^{{\left( {\frac{{ - E_{\upalpha} }}{RT}} \right)}} f(\alpha ), $$

where *α* is the degree of conversion at time *t* and *f* (*α*) indicate the reaction mechanism function.

The thermal decomposition is reflected by the conversion degree *α* which could be defined as follows:

4 $$ \alpha = \frac{{m_{0} - m_{t} }}{{m_{0} - m_{\infty } }}, $$

where *m*_0_, *m*_*t*_, and *m*_*∞*_ indicate the initial, instantaneous, and final masses during thermal degradation, respectively.

By understanding that temperature increases with respect to time under constant heating rate (*β*), *β* can be expressed as follows:

5 $$ \beta = \frac{{{\text{d}}T}}{{{\text{d}}t}} = \frac{{{\text{d}}T}}{{{\text{d}}\alpha }}{ \times }\frac{{{\text{d}}\alpha }}{{{\text{d}}t}}. $$

From Eqs. (4) and (2):

6 $$ \frac{{{\text{d}}\alpha }}{{{\text{d}}T}} = \frac{A}{\beta }e^{{\left( {\frac{{ - E_{\alpha } }}{RT}} \right)}} f(\alpha ). $$

The integrated form of *f* (*α*) can be expressed as follows:

7 $$ g(\alpha ) = \int_{0}^{\alpha } {\frac{{{\text{d}}\alpha }}{f(\alpha )}} = \frac{A}{\beta }\int_{{T_{0} }}^{T} e^{{\left( {\frac{{ - E_{a} }}{RT}} \right)}} {\text{d}}T. $$

An exact solution for the above integral cannot be obtained, and thus, Eq. (7) needs to be solved by employing approximations or numerical methods. The *iso*conversional methods, in view of their good adaptability and validity, are known to provide a viable method to identify the apparent activation energy. Thus, in the present study, two *iso*conversional methods Kissinger–Akahira–Sunose (KAS) and Flynn–Wall–Ozawa (FWO) methods are applied to determine the apparent activation energy.

The FWO method can be expressed as follows:

8 $$ \ln (\beta ) = \ln \left[ {\frac{{AE_{\alpha } }}{Rg(\alpha )}} \right]\, - 5.331 - 1.0516\frac{{E_{\alpha } }}{RT}. $$

The KAS method can be expressed as follows:

9 $$ \ln \left( {\frac{\beta }{{T^{2} }}} \right) = \ln \left[ {\frac{AR}{{E_{\alpha } g(\alpha )}}} \right] - \frac{{E_{\alpha } }}{RT}. $$

At constant conversion rate (*α*) and multiple heating rates, the plots ln (*β/T*^2^) vs. 1/*T* (KAS method) and ln (*β*) vs. 1/*T* (FWO method) result in straight lines, whose slope can be used to calculate the apparent activation energy. Generalized master plots’ methods was used to identify the reaction mechanism function *f*(*α*). Theoretical master plots, listed in Table 1, are considered as a reference and were compared against the experimental master plots. The underlying concept related to generalized master plots method is, at infinite temperature, the generalized time (*θ*) required to achieve a certain degree of conversion (*α*) [24, 25] can be given by the following equation:

10 $$ \theta = \int_{0}^{t} {e^{{\left( {\frac{ - E\alpha }{RT}} \right)}} } \,{\text{d}}t. $$

By differentiating the above equation:

11 $$ \frac{{{\text{d}}\theta }}{{{\text{d}}t}}\, = \,e^{{\left( {\frac{ - E\alpha }{RT}} \right)}} . $$

By combining (1), (2), and (10), we get the following:

12 $$ \frac{{{\text{d}}\alpha }}{{{\text{d}}\theta }}\, = \,\frac{{{\text{d}}\alpha }}{{{\text{d}}t}}\,e^{{\left( {\frac{ - E\alpha }{RT}} \right)}} . $$

We can relate a proper kinetic model, by selecting *α* = 0.5 as a reference, to reduced generalized rate of reaction as given by the following:

13 $$ \frac{{\frac{{{\text{d}}\alpha }}{{{\text{d}}\theta }}}}{{\left( {\frac{{{\text{d}}\alpha }}{{{\text{d}}\theta }}} \right)0.5}}\, = \,\frac{f(\alpha )}{f\left( \alpha \right)0.5}\, = \,\left( {\frac{{\frac{{{\text{d}}\alpha }}{{{\text{d}}\theta }}}}{{\left( {\frac{{{\text{d}}\alpha }}{{{\text{d}}\theta }}} \right)0.5}}} \right)\,\frac{{e^{{\left( {\frac{ - E\alpha }{RT}} \right)}} }}{{e^{{\left( {\frac{ - E\alpha }{RT0.5}} \right)}} }}. $$

Therefore, by plotting the generalized reaction rate from the right-hand side of Eq. (13), and the theoretical plots from the left-hand side of Eq. (13) against conversion (*α*), appropriate reaction mechanism function can be inferred on comparison.

### Compensation effect

While the model free methods are sufficient to determine apparent activation energy, compensation effect allows to accurately determine pre-exponential factor and reaction model. A strong correlation in the form of linear relationship between the Arrhenius parameters, ln *A*_*j*_ and *E*_*j*_ estimated using single heating rate method, is known as compensation effect. Taking logarithm on both sides and rearranging terms, Eq. (3) results in Eq. (14):

14 $$ \ln \left[ {\frac{1}{fi(\alpha )}\frac{{{\text{d}}\alpha }}{{{\text{d}}t}}} \right]\, = \,\ln Ai(\alpha ) - \,\frac{E\alpha (\alpha )}{RT}, $$

where *i* indicate the reaction model in Table 1.

Selecting any reaction model *f*_*i*_(*α*) mentioned in Table 1, and by plotting the left-hand side of Eq. (13) against the inverse of temperature, a pair of ln *A*_*i*_ and *E*_*αi*_ can be generated from the intercept and slope of straight line. Then the compensation equation is given as follows:

15 $$ \ln A_{i} \, = \,a^{*} E_{\alpha i} \, + \,b^{*} , $$

where a^*^ and b^*^ are generated from linear fitting of these pairs of ln *A*_*i*_ and *E*_*α*,*i*_. By substituting the activation energy obtained from model free isoconversional methods (Eq. 9) and a^*^, b^*^ values obtained from Eq. (14) in the following equation, the pre-exponential factor at given *α* can be calculated using the following equation:

16 $$ \ln A_{\alpha } = \, a^{*} E_{\alpha } + \, b^{*} , $$

where *E*_*α*_ was obtained from isoconversional methods. By rearranging Eq. (3), the reaction model *f*(*α*) can be defined as follows:

17 $$ f(\alpha )\, = \,\left( {\frac{{{\text{d}}\alpha }}{{{\text{d}}t}}} \right)_{\alpha } \,\left[ {A\alpha \exp \left( { - \,\frac{E\alpha }{RT}} \right)} \right]^{ - 1} . $$

By substituting the experimental values for (d*α*/d*t*) and *T*_*α*_ and using the values of activation energy and pre-exponential factor derived from isoconversional methods and compensation effect, respectively, Eq. (16) yields numerical values for *f*(*α*), which can be matched against theoretical *f*(*α*) models to identify the most accurate reaction model.

The thermodynamic parameters such as, free Gibbs energy (Δ*G*), enthalpy (Δ*H*), and change in entropy (Δ*S*) were evaluated using Eqs. (18) to (20) [26, 27]:

18 $$ \Delta G\,\, = \,\,E_{\alpha } + RT_{m} \ln \left[ {\frac{{k_{B} T_{m} }}{h\,A}} \right] $$

19 $$ \Delta H\,\, = \,\,E_{\alpha } - RT $$

20 $$ \Delta S\,\, = \frac{\Delta H - \Delta G}{{T_{m} }}, $$

where T_*m*_, *k*_*B*_, and *h* indicate DTG peak temperature, Boltzmann constant (1.381 × 10^−23^ J K^−1^), and Plank constant (6.626 × 10^−34^ J s), respectively.

## Results and discussion

### Thermal behavior of microalgae, digestate, and their blends

**Fig. 1:**
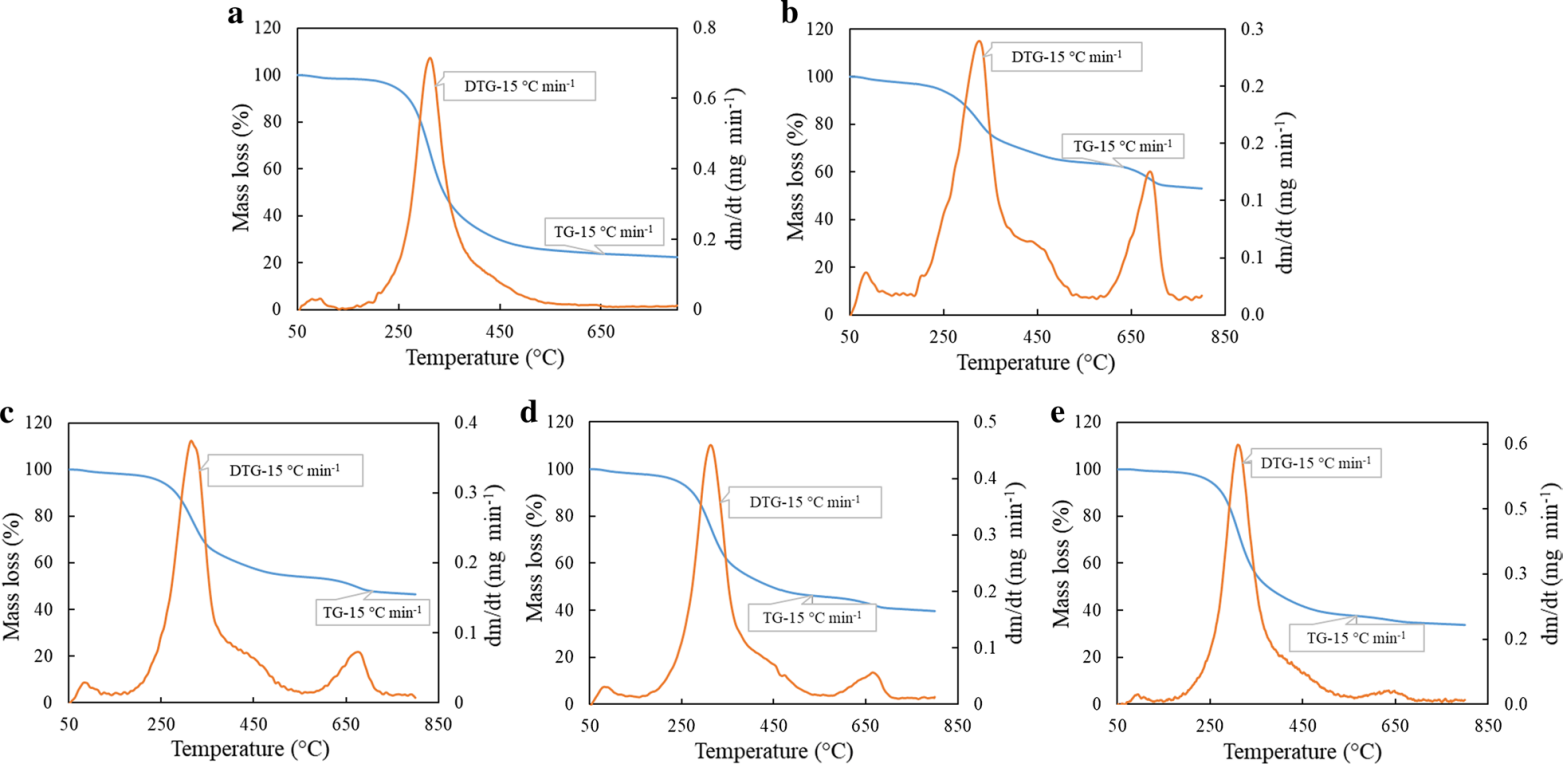
TG and DTG curves of biomass samples and their blends: **a** microalgae, **b** digestate, **c** MD-1, **d** MD-2, and **e** MD-3 at heating rate 15 °C min^−1^

The TG and DTG curves for the (co-)pyrolysis process for selected biomass samples, which include MA, SWD, and their blends MD-1 (25% MA + 75% SWD w/w) MD-2 (50% MA + 50% SWD w/w) and MD-3 (75% MA + 25% SWD w/w), generated at 15 °C min^−1^ heating rate, are presented in Fig. 1.

During pyrolysis, the organic components of biomass are decomposed into different vapor phases and gas compounds leaving a carbon-rich solid residue (pyrolysis char). The mass loss in samples during pyrolysis process is mainly because of evolution of vapor and gas from the biomass. The pyrolysis process of MA, SWD, and their blends (MD-1, 2, and 3) can be divided into three zones. In the first zone, the pyrolysis took place at low temperatures (< 200 °C), and this can be attributed to the evaporation of physically absorbed moisture [28]. The main pyrolysis of the plain samples, MA and SWD, took place between 200 and 500 °C, and are in good agreement with the other studies reported in the literature [29, 30]. The second zone could be considered as the main pyrolysis stage as maximum weight loss was noticed in this zone for the individual samples and their blends. For MA sample (Fig. 1a), the second zone occurred in the temperature range 200–600 °C with a characteristic peak at 315 °C, followed by a shoulder in between 400 and 500 °C. The main peak can be attributed to the decomposition of proteins. The maximum degree of weight loss was noticed at ca. 300–340 °C. As microalgae do not contain cellulose and hemicellulose, weight loss can be attributed to the decomposition of structural components such as lipids, lignin, proteins, and carbohydrates. In this temperature range, these compounds are reported to undergo a set of reaction mechanisms, which include decarboxylation, depolymerization, and cracking of primarily carbohydrates, lipids, and proteins [31]. The small shoulder at the end of the second zone could possibly be the result of lipids and proteins present in microalgae (Table 2).

On the other hand, the DTG profile for the pyrolysis of SWD showed three distinct peaks (Fig. 1b). The main degeneration stage was identified to be in the temperature range 200–500 °C. The main peak was noticed at ca. 330 °C and can be ascribed to the degradation of hemicellulose and glucoside linkage depolymerisation [30]. Swine manure cannot be considered as common lignocellulosic biomass as it not only contains cellulose, hemicellulose, and lignin, but also contains a large amount of extractives (such as sugars, proteins, lipids, starches, etc.). These extractives cannot be overlooked as they contribute significantly to the total weight of biomass and have a decomposition range matching closely that of hemicellulose [8]. A small shoulder at the right of the major peak can be ascribed to the decomposition either of lipids or of other *N*-containing compounds. In addition, a smaller peak close to 700 °C was witnessed and this could be because of the dehydration or calcination of mineral components [32]. The DTG profiles of MD-1 (Fig. 1c), MD-2 (Fig. 1d), and MD-3 (Fig. 1e) were similar to the biomass that contributed to the major portion of blend. For instance, the DTG profile of MD-1 is similar to that of SWD, while that for MD-3 the DTG profile was close to MAs. However, the peak temperatures of the blends decreased when the proportion of MA was increased. The peak temperatures for MD-1, 2, and 3 were 320, 316, and 314 °C, respectively, with the major weight loss for all the three blends happening in the temperature range of 270–370 °C. When the TGA data of the individual biomass (SWD) are compared against the blends of MA and SWD, no remarkable synergistic effect can be seen during co-pyrolysis, as the amount of solid residue left at the end of co-pyrolysis (at 800 °C) is an intermediate value between the residues during the pyrolysis individual biomasses. However, the residues obtained from the pyrolysis of blends were much lower than the SWD pyrolysis residue. This could be because of evolution of hot gaseous species as a result of thermal decomposition of volatile matter or could be because of thermal decomposition of biomass enhanced by catalytic activity of metal contents in the MA and SWD ash [33].

### Analysis of evolved gas species

**Fig. 2:**
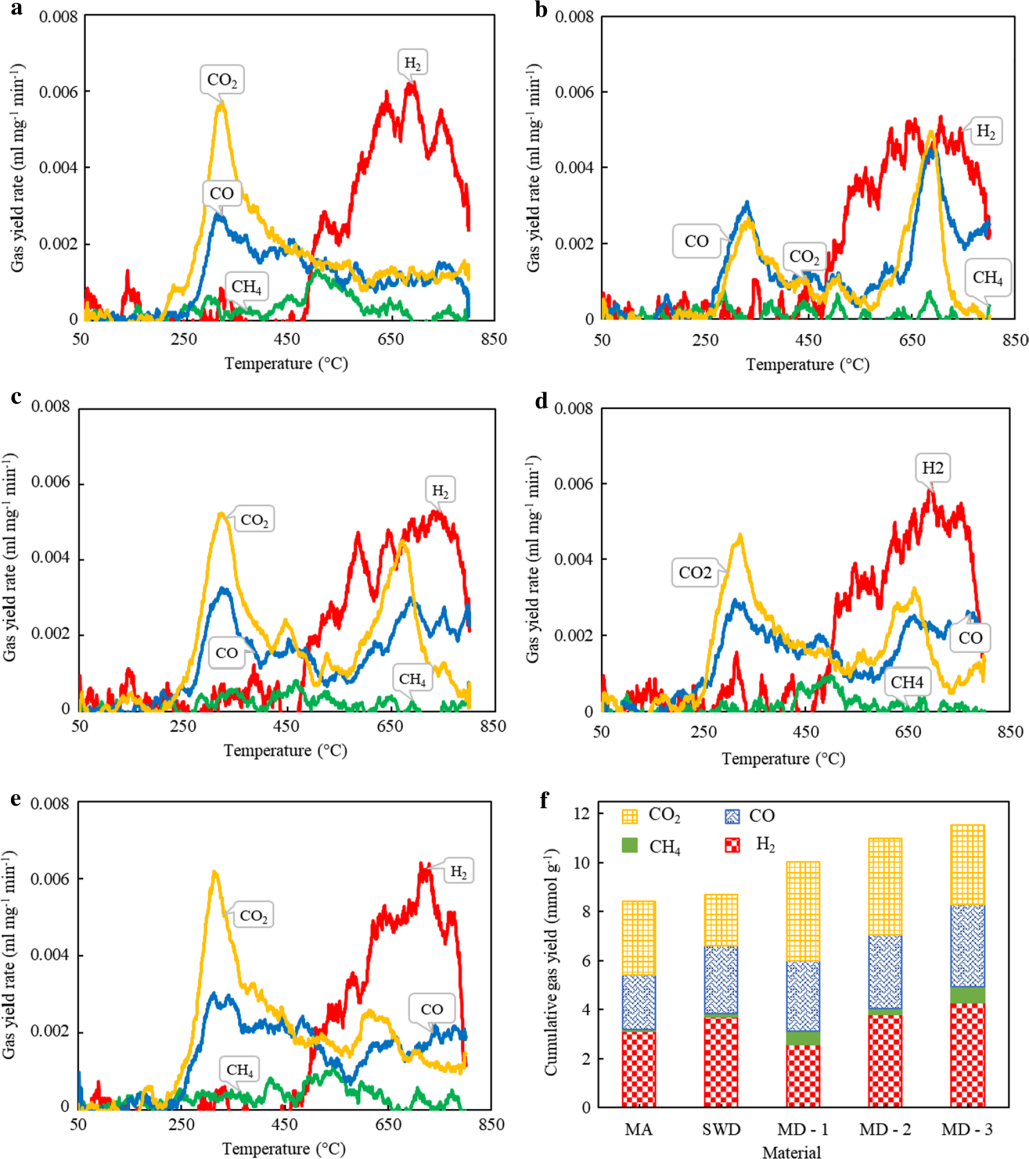
Gas yield rates for samples: **a** MA **b** SWD, **c** MD-1, **d** MD-2, **e** MD-3, and **f** cumulative gas yields for all the samples

The gas evolution trends for all the samples are depicted in Fig. 2. There were two distinct phases of gas evolution from pyrolysis. The first gas release coincided with the main pyrolysis weight loss, and the released gases, primarily CO and CO_2_, evolved within the temperature range of 250–400 °C. At temperatures 450 °C and above, H_2_ released due to tar cracking and reforming reactions was noted and it continued up to 800 °C. There was very little CH_4_ released during the pyrolysis of all the samples. These appeared to be the general trends of pyrolysis which were shared across all the samples, however, to intensities of gas releases seemed to differ across samples. For MA (Fig. 2a), two CO_2_ evolution peaks, one at 220 °C and the other at 324 °C, were noticed. These peaks can be associated with the decomposition of saccharides and main decomposition of proteins and lipids [34]. For SWD (Fig. 2b), two distinct CO_2_ evolution peaks were noticed at 333 °C and 688 °C. These can be attributed to the decomposition of volatiles and solid residue respectively, as observed in thermogravimetric analysis of SWD. For the blends (Fig. 2c-MD-1-, Fig. 2d-MD-2, Fig. 2e-MD-3), with the increase in MA content, the second peak value of CO_2_ decreased gradually. Similar behavior was observed during the co-pyrolysis of microalgae with textile dying sludge [35]. It can be noticed from Fig. 2 that the CO evolution trend appeared to be similar for all the samples. The evolution of CO started from temperatures ca. 200 °C and continued thorough out the run. The possible reasons could be decomposition of volatiles and Boudouard reactions occurring in temperatures < 500 °C and 500–800 °C, respectively. For blends, the second evolution peak for CO followed similar trend as seen for CO_2_ and the second peak gradually decreased as the proportion of MA grew. The trends from MA and SWD, Fig. 2a, b, showed a similar H_2_ release, but their CO and CO_2_ releases differed. However, there was a mutually synergistic effect on total gas yield between the two biomass types. It can be noticed from Fig. 2f that the gas yields increased with the rise in the composition of MA in the blends. In addition, the total gas yield was obtained in the order MD-3 > MD-2 > MD-1 > SWD > MA. The plausible reasons for an increase in individual gas compositions as well as total gas yield could be char gasification reactions and reforming reactions. In addition, the alkali metals present in the ash of MA and SWD biomasses could have improved the yield rates and consequently also the final yields.

### Evidence of synergy during pyrolysis of biomass blends

To evaluate quantitatively the possible synergistic effects during the co-pyrolysis of biomass blends, the experimental results (TGA data) were compared with the calculated results, i.e., weighted residual mass values (additive model), if the biomasses had been pyrolysed independently. This is equivalent to a complete lack of synergistic interaction between the two biomass samples during pyrolysis, so that the calculated values are the sum of individual values proportional to their mass ratio. More detailed explanation regarding the procedure can be obtained from the work done by Mallick et al. [33], The weighted residual mass values as functions of time can be obtained from the following equation:

21 $$ \alpha_{\text{cal}} \, = \,(1 - f_{\text{MA}} )\;\alpha_{\text{SWD}} \, + \,f_{\text{MA}} \,\,\alpha_{\text{MA}} , $$

where *f*_MA_ is the fraction of microalgae in the mixture, and *α*_MA_ and *α*_SWD_ are the conversions of individual biomasses at a given time. Hence, if individual biomasses are pyrolysed independently, *α*_cal_ can be defined as theoretical conversion of a given biomass blend with zero synergy.

**Fig. 3:**
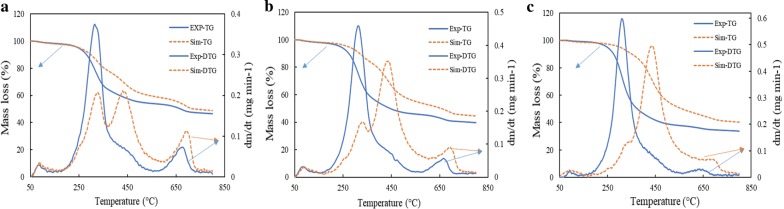
Comparison of experimental and calculated TGA profiles of biomass for heating rate of 15  °C min^−1^: **a** MD-1, **b** MD-2, and **c** MD-3

Using Eq. (21), the theoretical conversions of biomass blends, calculated by using the TGA data of individual biomasses, are compared with the experimental data and the results are presented in Fig. 3a–c. A significant discrepancy can be noticed between the experimental and calculated profiles of blends MD-1 and MD-2 and MD-3, especially in the temperature range of 250–650 °C. The experimental thermal degradations of all the blends were more extensive than the calculated additive degradations of individual biomasses of same mass ratio, and this essentially is the evidence of mutual synergy for thermal degradation between the two biomasses and is discussed in greater details in the subsequent sections.

### Kinetic analysis

#### Estimation of apparent activation energy of thermal degradation

**Fig. 4:**
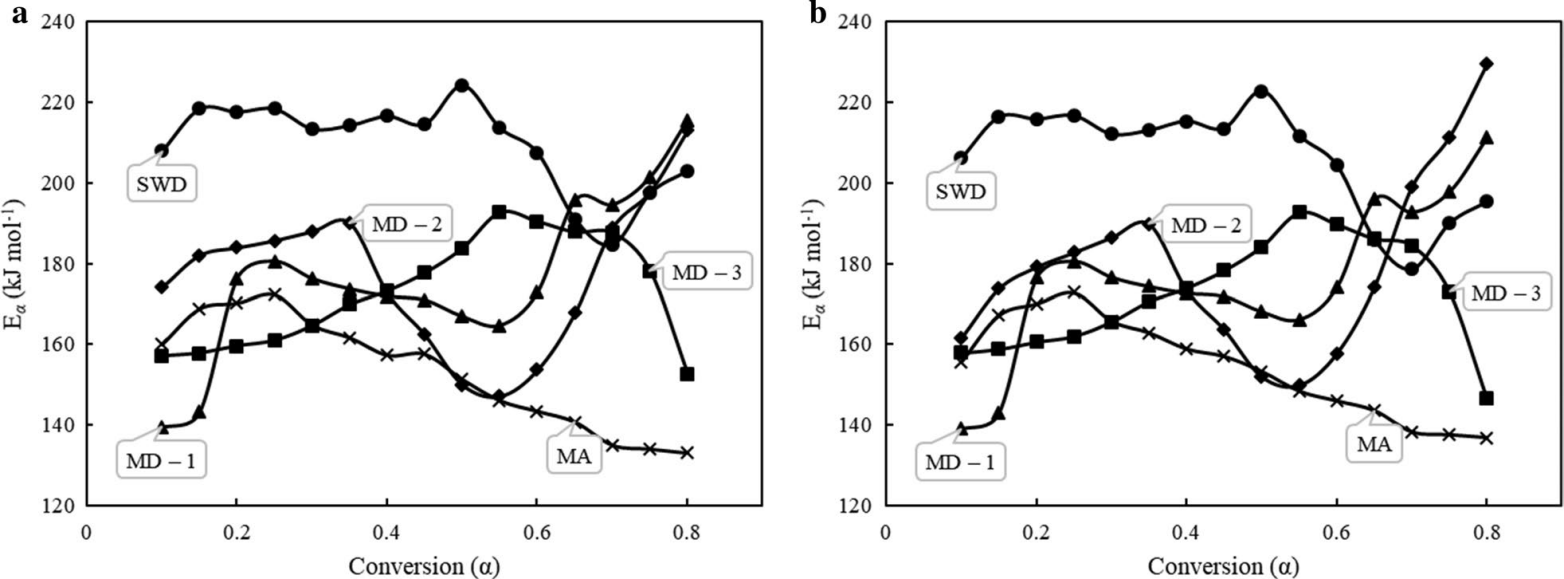
Variation in activation energy with progressing conversion for **a** using KAS and **b** using FWO models

Thermogravimetric data was used to analyze the kinetic parameters of the samples and blends that are considered in the present study. The description of transport phenomena together with chemical kinetics is crucial in the design and optimization of thermochemical conversion systems [36]. Considering the TGA data obtained at heating rates 10, 15, and 20 °C min^−1^ and using the isoconversional methods, namely KAS and FWO methods, the apparent activation energies were determined for the materials used in the study. According to Eqs. (7) and (8), the apparent activation energies were calculated within a selected conversion range of 0.1–0.8 with an interval of 0.05, and are presented in Table 5. Activation energy is defined as the minimum energy required to initiate a reaction, which imply that a reaction with higher activation energy either need higher reaction temperature or longer duration to gain adequate energy to initiate the reaction [37]. When many reactions are present, an apparent activation energy is derived from the data, representative of the ensemble of reactions taking place, as is the case for pyrolysis. The change in the apparent activation energy with respect to conversion is presented in Fig. 4. From Table 5 and Fig. 4, it can be noticed that apparent activation energy (*E*_*α*_) is highly dependent on conversion which indicates that pyrolysis of biomass is, as expected, a complex process involving many reactions occurring simultaneous at the same stage [38]. The overall pyrolysis process can be characterized as a multi-stage reaction in which every single stage contributes to global mechanism to some extent depending on the decomposition. The average activation energies for samples and their blends using KAS and FWO methods were in the range of 153.17–157.55 for MA, 206.45–209.51 for SWD, 176.07–176.25 for MD-1, 177.13–178.99 for MD-2, and 172.29–172.94 for MD-3. The activation energies obtained in this study are close to the values reported in the literature for similar feedstock. For instance, Shuping et al. [39] and Fernandez-Lopez et al. [40] reported an average range for *E*_*α*_ of 145.71–146.42 and 210–213 for microalgae *Dunaliella tertiolecta* and swine manure, respectively, using KAS and FWO methods.

**Table 5:** Activation energies (*E*_*a*_) and correlation factors (*R*^2^) for different conversion values using KAS and FWO models

Material	MA				SWD				MD-1				MD-2				MD-3			
Method	KAS		FWO		KAS		FWO		KAS		FWO		KAS		FWO		KAS		FWO	
Parameter/conversion	*E*_*a*_^a^	*R* ^*2*^	*E*_*a*_^a^	*R* ^*2*^	*E*_*a*_^a^	*R* ^*2*^	*E*_*a*_^a^	*R* ^*2*^	*E*_*a*_^a^	*R* ^*2*^	*E*_*a*_^a^	*R* ^*2*^	*E*_*a*_^a^	*R* ^*2*^	*E*_*a*_^a^	*R* ^*2*^	*E*_*a*_^a^	*R* ^*2*^	*E*_*a*_^a^	*R* ^*2*^
0.1	160.07	0.9773	155.65	0.9731	207.91	0.9997	206.07	1	139.45	0.9951	139.05	0.997	174.05	0.9946	161.61	0.9864	157.09	0.9976	157.9	0.9978
0.15	168.9	0.9999	167.24	0.997	218.38	0.9947	216.26	0.995	143.25	0.9987	143.02	0.9994	182.04	0.9998	173.82	0.9981	157.83	0.994	158.79	0.995
0.2	170.26	0.9949	170.07	0.9958	217.63	1	215.75	1	176.27	0.9805	176.46	0.9823	183.92	0.9989	179.15	0.9998	159.6	0.9973	160.6	0.9976
0.25	172.33	0.9935	172.83	0.9941	218.35	0.9994	216.59	1	180.5	0.9909	180.59	0.9917	185.68	0.9945	182.74	0.9964	160.97	0.9976	162.01	0.9978
0.3	164.85	0.9955	165.8	0.996	213.4	0.9995	212.02	1	176.22	0.9987	176.64	0.9988	187.95	0.989	186.48	0.991	164.62	0.9985	165.58	0.9987
0.35	161.74	0.9977	162.93	0.9979	214.27	0.9997	212.97	1	173.7	0.9999	174.35	1	190.02	0.9886	189.82	0.9869	169.83	0.9971	170.62	0.9974
0.4	157.41	0.9985	158.89	0.9987	216.54	1	215.23	1	171.9	0.9986	172.73	0.9987	172.74	0.9972	173.47	0.9975	173.19	0.9998	173.89	0.9986
0.45	157.63	0.9993	157.05	0.9994	214.62	0.9996	213.51	1	170.94	0.9903	171.91	0.9914	162.4	1	163.73	1	177.71	0.9986	178.27	0.9988
0.5	151.38	0.9998	153.31	0.9998	224.18	1	222.71	1	166.88	0.9812	168.15	0.9844	150.03	0.9965	152.08	0.9969	183.84	0.9981	184.18	0.9983
0.55	146.13	0.9998	148.4	0.9998	213.62	0.9922	211.67	0.992	164.73	0.9648	166.21	0.9686	147.18	0.9909	149.98	0.9925	192.75	0.9976	192.62	0.9979
0.6	143.47	0.9992	145.97	0.9993	207.49	0.9887	204.3	0.989	172.9	0.9764	174.14	0.9811	153.68	0.991	157.84	0.9936	190.46	0.9993	189.71	0.9992
0.65	140.8	0.998	143.55	0.9982	190.96	0.9839	185.91	0.982	195.6	0.9782	195.98	0.9844	167.78	0.99	174.25	0.9945	187.85	0.9969	186.16	0.997
0.7	135.05	0.9955	138.24	0.9961	184.76	0.9846	178.52	0.982	194.54	0.9714	192.79	0.9809	188.77	0.9881	199.03	0.9953	187.63	0.999	184.44	0.9989
0.75	134.11	0.9956	137.53	0.9962	197.49	0.9943	189.9	0.988	201.35	0.9906	197.83	0.9961	197.77	0.9854	211.41	0.9952	178.17	0.9954	173.03	0.9946
0.8	133.1	0.9963	136.82	0.9969	203.04	0.9796	195.27	0.977	215.48	0.9906	211.21	0.9962	212.99	0.9716	229.47	0.9879	152.63	0.9999	146.53	1
Average	153.15	0.9961	154.25	0.9959	209.51	0.9944	206.45	0.994	176.25	0.9871	176.07	0.9901	177.13	0.9917	178.99	0.9941	172.94	0.9978	172.29	0.9978
Calculated *E*_*a*_	–		–		–		–		195.42		193.4		181.33		180.35		167.24		167.3	

^a^In kJ mol^−1^

The *E*_*α*_ were noticed to increase from *α* = 0.15 which indicate the pyrolysis of main components of biomass (lipid, protein, and carbohydrate in MA and cellulose, hemicellulose, and lignin in SWD). With the increase in the conversion, the pyrolysis of biomasses and their blends can be roughly categorized into different stages (depending on the biomass and their blending ratio). For MA, the pyrolysis with respect to conversion can be divided into two stages: *α* = 0.1–0.25 and *α* = 0.25–0.8. The temperatures corresponding to stages I and II are 250–300 and 300–500 °C, respectively. During stage I, the activation energy increased from 160 to 172 kJ mol^−1^. As per the previous literature and DTG curves, discussed above in this study, carbohydrates and proteins of microalgae biomass decomposed in this stage. In the stage II, the activation energy decreased continuously from 164.8 kJ mol^−1^ at *α* = 0.3 to 133.1 kJ mol^−1^ at *α* = 0.8. For SWD biomass and blends, MD-1 and MD-2, the pyrolysis process, with respect to conversion, can be divided into three stages. Based on the DTG analysis in this study, it can be inferred that most of the extractives (lipids, proteins, and carbohydrates) and hemicellulose decomposed in stage I, which varied from *α* = 0.1–0.25 for SWD and MD-1 and *α* = 0.1–0.35 for MD-2. The difference in the range for MD-1 and MD-2 could be the variation in the proportion of biomasses. In stage I, the E_*α*_ values increased from 207.9 to 218.3, 139.5 to 180.3, and 174 to 190 kJ mol^−1^ for SWD, MD-1, and MD-2, respectively. In stage II, for SWD, the activation energy almost kept a constant value for *α* varying from 0.25 to 0.55, while the activation energy decreased continuously from 171 to 164 and 172–153 kJ mol^−1^ for blends MD-1 and 2, respectively. Finally, the activation energies climbed up in stage III as shown in Fig. 4. It should be noted that the behavior of blends is similar to the individual mass-dominant biomass. For MD-3 the trend of activation energy against conversion is similar to MAs. The overall pyrolysis for MD-3 can be divided into two stages, where, in stage I, the activation energy increases continuously to 192.7 kJ mol^−1^ at *α* = 0.55 and then decreases to 146.5 kJ mol^−1^ at *α* = 0.8. The synergistic effect in co-pyrolysis of biomass is evident in the terms of activation energy. It can be witnessed from Table 4 that average activation energies of blends MD-1 and 2 have a synergy of SWD with respect to MA in up to 50 w/w %, as the calculated weighted blend activation energies (0 synergy) were higher than those found experimentally. All the blends displayed a higher activation energy than MAs, revealing lack of mutual synergy. The plausible reason could be that the extractives present in MA biomass may not have enhanced the degradation of structural components in SWD biomass, majorly lignin, cellulose, and hemicellulose. Furthermore, the synergistic effect of additional heating due to volatiles content is also limited as the SWD biomass contain lower volatiles when compared with other forms of biomass such as lignocellulosic biomass [33]. As a result, the activation energy of blends is marginally higher than that of MA biomass. However, a synergy can be noticed as the average activation energy of blends is significantly lower than pure SWD biomass.

#### Evaluation of pre-exponential factor and other thermodynamic parameters

**Fig. 5:**
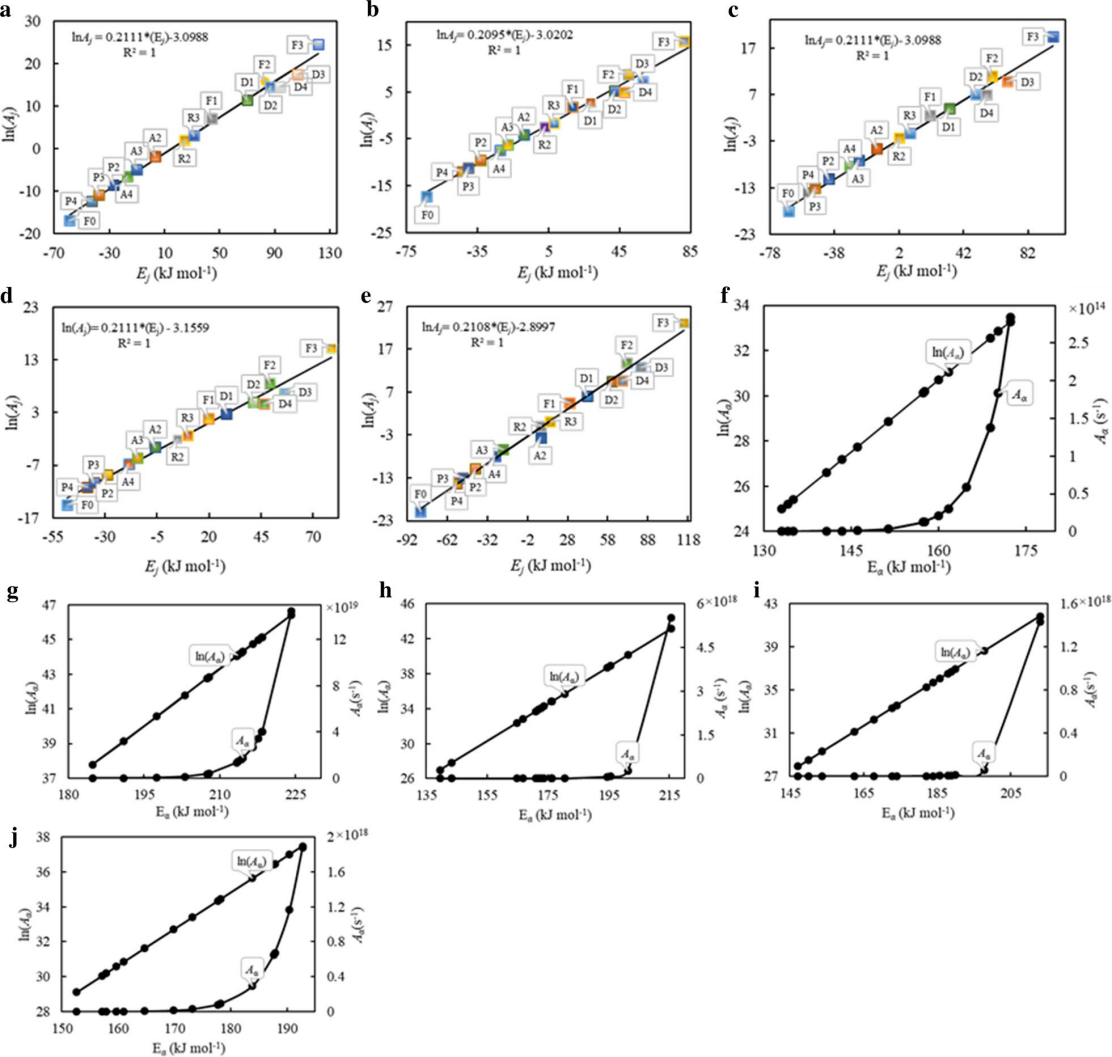
The compensation line of Arrhenius parameters for **a** MA, **b** SWD, **c** MD-1, **d** MD-2, and **e** MD-3; ln*A*_*α*_ vs. *E*_*α*_ dependencies and *A*_*α*_ vs. *E*_*α*_ dependencies for **f** MA, **g** SWD, **h** MD-1, **i** MD-2, and **j** MD-3

The compensation effect was used to estimate the pre-exponential factor by using Eqs. (15) and (16), while the other thermodynamic parameters, such as activation enthalpy, activation entropy, and Gibbs free energy, were identified using Eqs. (18)–(20). Using the activation energy obtained using FWO method, pre-exponential factor can be obtained from the compensation line. Considering each *f*_*i*_(*α*) from Table 1 into Eq. 14, 15 pairs of ln *A*_*i*_ and *E*_*α,i*_ are obtained and are plotted in Fig. 5a–e. The variation of ln (*A*_*α*_) and *A* (s^−1^) with respect to *E*_*α*_ is presented in Fig. 5f–j for MA, SWD, MD-1, MD-2, and MD-3, respectively. The values of pre-exponential factor and other thermodynamic parameters for biomasses MA, SWD, and for blends MD-1, 2, and 3 are listed in Table 6. The pre-exponential factor (*A*) showed variation in a wide range from 10^10^ to 10^20^ (depending on material and blending ratio) over the conversion range 0.1–0.8. This reflects the complex nature of biomasses and of the reactions that occur during the process of pyrolysis. The *A* values ≤ 10^9^ s^−1^ indicate surface reactions, but, if the reactions are not dependent on the surface area, a low *A* may also indicate a closed complex (tight junctional complex), while values above 10^9^ s^−1^ indicate a loose junctional complex [36, 41]. For *A* ranging in between 10^10^ and 10^12^ s^−1^, when compared to the initial reagent, the activated complex was restricted in rotation [42]. In case of unimolecular materials, the complex is further expected to interact more intensely with its neighbours by expanding its size. From Table 5, within the decomposition range of lignin component (*α* = 0.5–0.8), the values of *A* of more than 10^14^ s^−1^ indicate a slower and more difficult degradation effect and imply the need of higher molecular collision. In such case, the reaction demands more energy and this scenario is in agreement with the activation energy characteristics (Table 5).

**Table 6:** Kinetic and thermodynamic parameters of thermal degradation of MA, SWD, MD-1, MD-2, and MD-3 under the heating rate (*β*) 15 °C min^−1^

Material	Parameter/conversion	Activation energy, *E*_*α*_ (kJ mol^−1^)	Pre-exponential factor, *A* (s^−1^)	Enthalpy, ∆*H* (kJ mol^−1^)	Gibbs free energy, °∆*G* (kJ mol^−1^)	Entropy, °∆*S* (J mol^−1^)
MA	0.10	155.6	7.19 × 10^10^	155.6	185.1	− 50.37
	0.15	167.2	8.91 × 10^10^	164.3	192.9	− 48.76
	0.20	170.1	1.09 × 10^11^	165.6	193.3	− 47.23
	0.25	172.8	3.65 × 10^11^	167.6	189.4	− 37.24
	0.30	165.8	6.41 × 10^11^	160.1	179.2	− 32.63
	0.35	162.9	1.12 × 10^12^	156.9	173.4	− 28.04
	0.40	158.9	3.41 × 10^12^	152.6	163.6	− 18.88
	0.45	157.1	1.22 × 10^13^	152.7	157.6	− 8.37
	0.50	153.3	1.28 × 10^13^	146.5	151.2	− 8.05
	0.55	148.4	2.13 × 10^13^	141.1	143.4	− 3.85
	0.60	146.0	3.04 × 10^13^	138.4	139.0	− 1.00
	0.65	143.6	5.86 × 10^13^	135.7	133.2	4.35
	0.70	138.2	1.38 × 10^14^	129.9	123.2	11.32
	0.75	137.5	1.84 × 10^14^	128.8	120.9	13.55
	0.80	136.8	2.84 × 10^14^	127.7	117.8	16.95
SWD	0.10	206.1	4.08 × 10^18^	203.5	144.2	98.21
	0.15	216.3	4.05 × 10^19^	213.8	143.1	117.1
	0.20	215.7	3.44 × 10^19^	213.0	143.2	115.6
	0.25	216.6	4.03 × 10^19^	213.6	143.1	116.7
	0.30	212.0	1.36 × 10^19^	208.6	143.6	107.6
	0.35	213,0	1.65 × 10^19^	209.4	143.5	109.1
	0.40	215.2	2.71 × 10^19^	211.6	143.3	113.1
	0.45	213.5	1.78 × 10^19^	209.6	143.5	109.5
	0.50	222.7	1.45 × 10^20^	219.2	142.5	126.9
	0.55	211.7	1.43 × 10^19^	208.5	143.6	107.5
	0.60	204.3	3.72 × 10^18^	202.4	144.2	96.20
	0.65	185.9	9.91 × 10^16^	185.7	145.9	65.93
	0.70	178.5	2.55 × 10^16^	179.4	146.5	54.43
	0.75	189.9	4.15 × 10^17^	192.0	145.2	77.36
	0.80	195.3	1.4 × 10^18^	197.3	144.7	87.15
MD-1	0.10	139.1	5.2 × 10^11^	134.6	154.6	− 33.88
	0.15	143.0	1.17 × 10^12^	138.4	154.6	− 27.34
	0.20	176.5	1.13 × 10^14^	171.8	165.6	10.53
	0.25	180.6	1.78 × 10^14^	175.9	167.5	14.22
	0.30	176.6	4.22 × 10^14^	171.9	159.2	21.31
	0.35	174.3	5.19 × 10^14^	169.5	156.0	22.93
	0.40	172.7	6.41 × 10^14^	167.8	153.3	24.61
	0.45	171.9	7.61 × 10^14^	167.0	151.6	25.96
	0.50	168.1	1.3 × 10^15^	163.2	145.2	30.34
	0.55	166.2	1.32 × 10^15^	161.2	143.2	30.34
	0.60	174.1	3.23 × 10^15^	169.1	146.7	37.73
	0.65	196.0	6.41 × 10^16^	190.8	153.8	62.47
	0.70	192.8	8.03 × 10^16^	187.7	149.6	64.18
	0.75	197.8	2.73 × 10^17^	192.5	148.6	74.13
	0.80	211.2	5.53 × 10^18^	205.7	147.1	98.83
MD-2	0.10	161.6	1.33 × 10^12^	157.1	172.5	− 26.08
	0.15	173.8	2.42 × 10^12^	169.2	181.8	− 21.41
	0.20	179.1	5.23 × 10^12^	174.5	183.3	− 15.10
	0.25	182.7	3.3 × 10^13^	178.0	177.9	0.21
	0.30	186.5	1.03 × 10^14^	181.7	176.1	9.65
	0.35	189.8	2.93 × 10^14^	185.0	174.3	18.33
	0.40	173.5	3.85 × 10^14^	168.6	156.6	20.56
	0.45	163.7	2.08 × 10^15^	158.8	138.6	34.61
	0.50	152.1	3.1 × 10^15^	147.1	124.9	37.86
	0.55	150.0	4.49 × 10^15^	145.0	121.0	40.88
	0.60	157.8	7.25 × 10^15^	152.8	126.5	44.80
	0.65	174.2	8.62 × 10^15^	169.1	142.1	46.15
	0.70	199.0	1.12 × 10^16^	193.8	165.6	48.21
	0.75	211.4	5.76 × 10^16^	206.1	169.9	61.69
	0.80	229.5	1.43 × 10^18^	224.0	172.2	88.32
MD-3	0.10	158.0	4.45 × 10^12^	153.4	162.8	− 16.06
	0.15	159.0	1.13 × 10^13^	154.2	159.2	− 8.51
	0.20	161.0	1.31 × 10^13^	156,0	160.3	− 7.35
	0.25	162.0	1.90 × 10^13^	157.3	159.9	− 4.40
	0.30	165.6	2.52 × 10^13^	160.8	162.1	− 2.10
	0.35	170.6	5.39 × 10^13^	165.8	163.4	4.13
	0.40	173.9	1.59 × 10^14^	169.0	161.4	13.08
	0.45	178.3	3.2 × 10^14^	173.4	162.3	18.81
	0.50	184.2	8.21 × 10^14^	179.3	163.6	26.56
	0.55	192.6	9.03 × 10^14^	187.64	171.6	27.27
	0.60	189.7	2.94 × 10^15^	184.7	163.0	36.99
	0.65	186.2	6.46 × 10^15^	181.1	155.6	43.44
	0.70	184.4	6.76 × 10^15^	179.3	153.6	43.68
	0.75	173.0	1.16 × 10^16^	167.7	139.6	48.01
	0.80	146.5	1.87 × 10^16^	141.1	110.7	51.73

From Table 6, it is evident that the change in activation enthalpy is in good agreement with the activation energy. It should be noted that, when the difference between *E*_*α*_ and ∆*H* is minimum, it indicates favorable conditions for the formation of activated complex [43]. From Table 5, when the *E*_*α*_ and ∆*H* values are compared against each other, a small energy barrier (~ 5 kJ mol^−1^) indicates the ease of reaction happening under the mentioned conditions. The changes in the Gibbs free energy (Δ*G*) indicate the increase in the total energy of the reaction system during the formation of activated complex. The positive values of Δ*G* indicate unfavorable conditions and requirement of excess energy as input [33]. The Δ*G* values were positive for all the materials and the blends considered in this study. Furthermore, the negative values of Δ*S*, as well as ΔG values higher than Δ*H* indicate that a significant amount of heat supplied to the system is unused or free. Entropy (Δ*S*) is commonly interpreted as the degree of disorder of the system. A small activation entropy indicates that the material is brought to a new state near its own thermal equilibrium after it has been through some kinds of physical or chemical aging phenomena. On the other hand, high values of activation entropy indicate that the material is far from its own thermal equilibrium. In the former case, the material shows little reactivity, which necessitates an increase in reaction time to form an activated complex. In the latter case, the material shows high reactivity and requires shorter reaction time to form an activated complex [42]. As can be seen in Table 6, the Δ*S* values for MD-1, 2, and 3 increased from − 33.9 to 98.8, − 26.1 to 88.3, and − 16.1 to 51.7 J mol^−1^, indicating an increase in the reactivity of the system.

#### Evaluation of reaction model

**Fig. 6:**
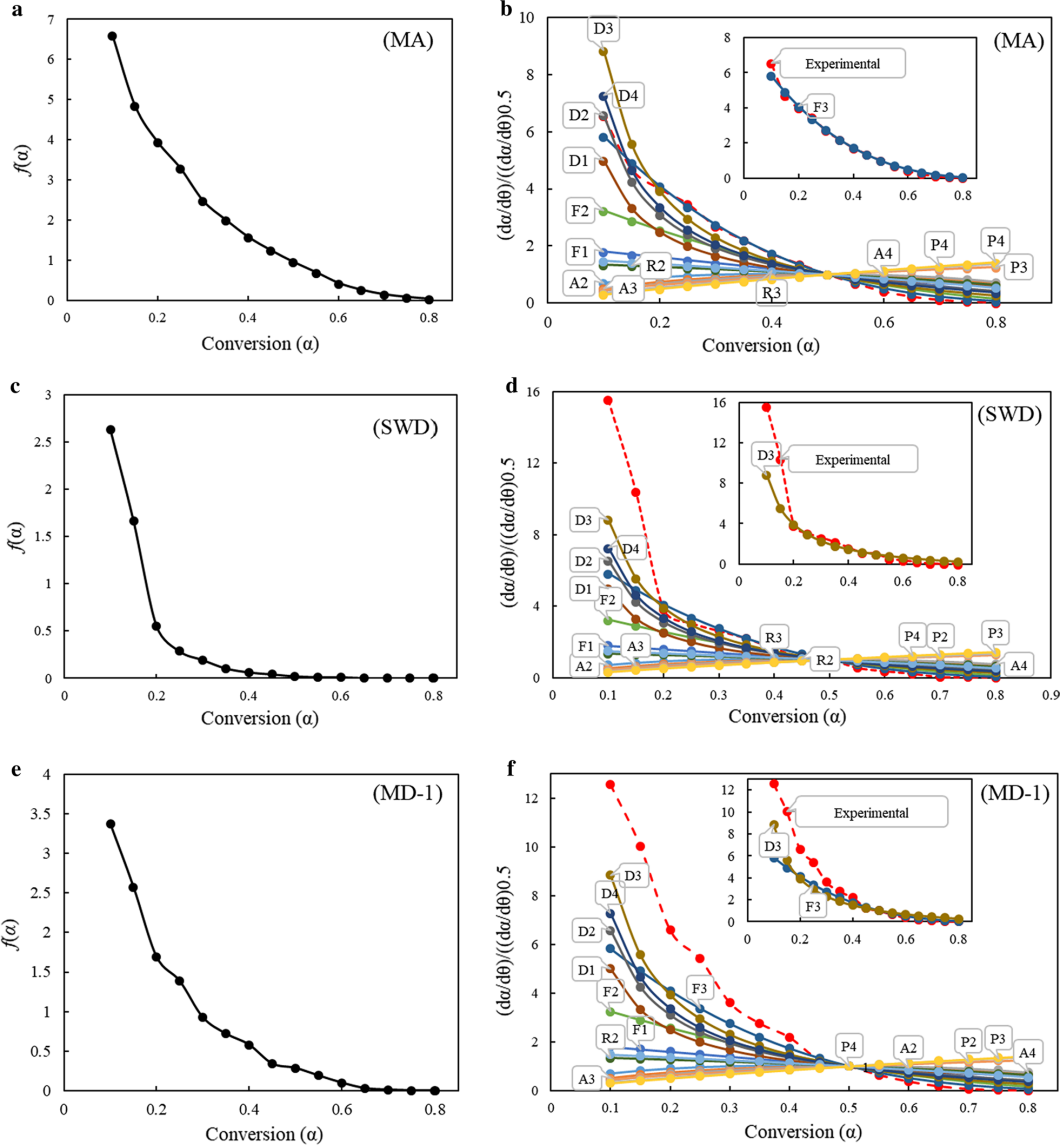

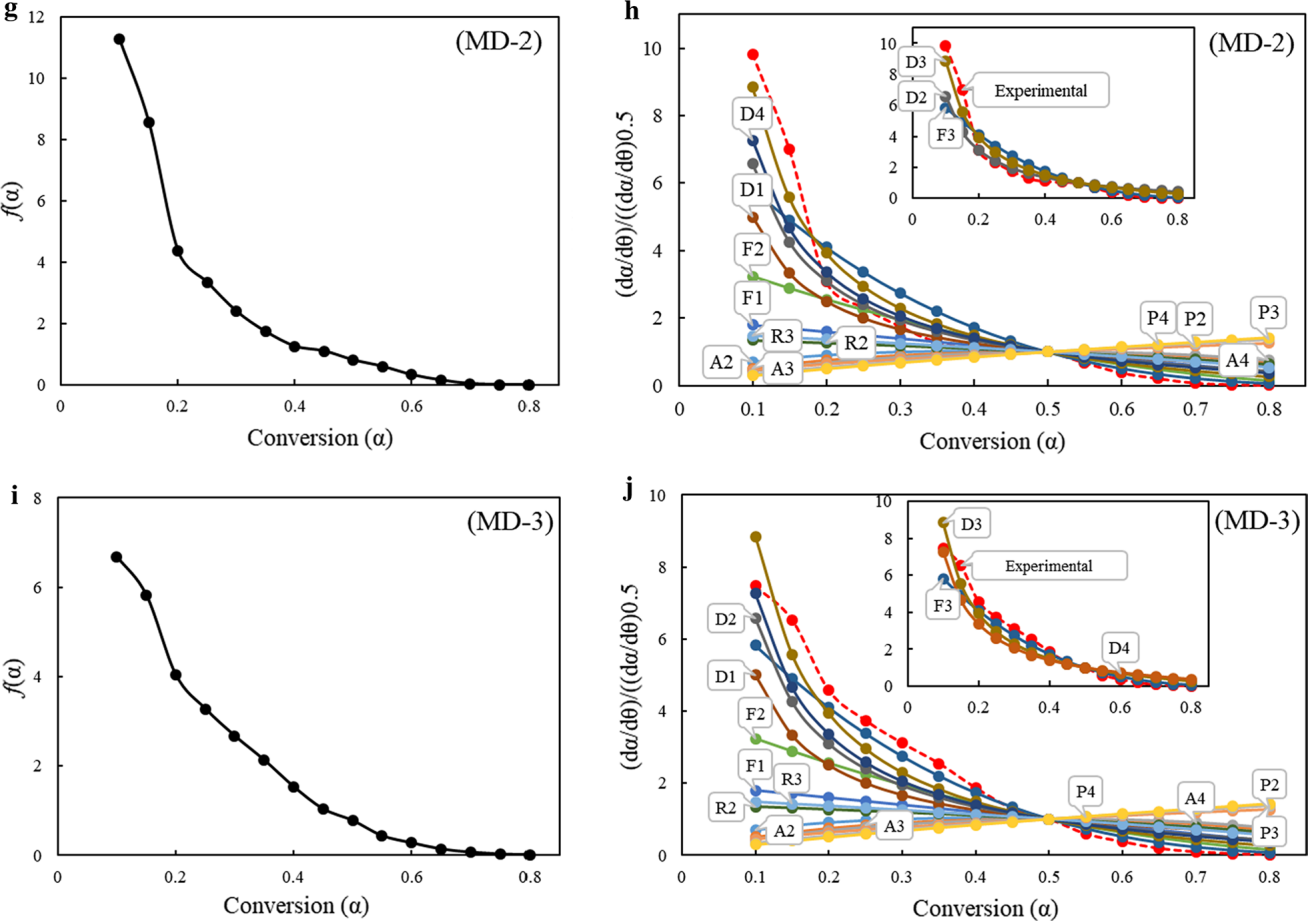
Reaction mechanisms for samples obtained (**a**), (**c**), (**e**), (**g**) and (**i**) using compensation effect (on the left) and (**b**), (**d**), (**f**), (**h**) and (**j**) using generalized master plots method (on the right side)

The generalized master plots are strictly influenced by the kinetic model used to fit the reaction but not by the heating rates. Therefore, in principle, the experimental master plots should take similar shapes for any heating rate. Using Eq. (13), experimental and theoretical master plots for different kinetic models mentioned in Table 1 were compared against each other. In addition, after identifying the pre-exponential factor (*A*) and apparent activation energy (*E*_*α*_), the *f*(*α*) function is numerically evaluated by using (17). The *f*(*α*) functions deduced from compensation effect and generalized master plots method for the materials studied are presented in Fig. 6, MA (Fig. 6a, b), SWD (Fig. 6c, d), MD-1 (Fig. 6e, f), MD-2 (Fig. 6g, h), and MD-3 (Fig. 6i, j). Though there are a number of reaction models available (Table 1), they can be categorized into three major groups describing the rate of change of conversion with temperature; accelerating, decelerating, and sigmoidal. The *f*(*α*) deduced from compensation effect can be used to categorize the reaction model into any of these three categories, while generalized master plots methods can be used to match experimental *f*(*α*) curves to theoretically available models. For sample MA, the experimental reaction model deduced using compensation effect indicates *f*(*α*) as a monotone function decreasing continuously with conversion. Based on the progression of *f*(*α*) with respect to conversion, *f*(*α*) can be classified into order-based models. This finding is in accordance with the curve obtained using the master plots method. The experimental curve closely matches with random nucleation with three nuclei on the individual particle (F3) reaction model (Fig. 6a, b). Similar trend was witnessed for SWD biomass sample, where the *f*(*α*) generated by using compensation effect was found to decrease continuously with the extent of conversion. This type of behavior can be related to reaction-order models or diffusion models.

It can be understood from Fig. 6d, that the *f*(*α*) function followed three-dimensional diffusion (Jander equation) (D3) in the entire conversion range. For blends MD-1 and MD-2, the experimental curves closely matched to D3 mechanism at lower conversions (*α* < 0.5) and then shifted to F3 mechanism at higher conversions. The reaction model of MD-3 was similar to that of MA biomass for conversions > 0.2. However, at lower conversions (*α* < 0.2), the blend MD-3 was in between D3 and three-dimensional diffusion (Ginstling–Brounshtein equation) (D4) mechanisms. Intuitive reasons why the 3D nucleation and nuclei growth model provided a better fit with MA samples, while the 3D diffusion model fitted best the SWD samples, can be supported by their different compositions and structures. Lack of lignin/cellulose/hemicellulose in MA samples resulted in a faster decomposition concurrent with low temperature evolution of CO and CO_2_. In contrast, SWD with cellulose (potentially crystalline), hemicellulose, and lignin which are harder to degrade, would need to overcome solid diffusion barriers, resulting in a wider spread evolution of the CO and CO_2_ gas products towards the higher temperatures. Decarboxylation and decarbonylation would occur more readily via nuclei and nuclei growth mechanism on the easily accessed solid reagents in the absence of strong fibre components, such as cellulose, hemicellulose, and lignin. The synergy would be reflected in a larger influence of 3D nucleation and nuclei growth than expected, and lowered diffusion barriers consistent with ash catalyzed reactions.

## Conclusions

The present study attempts to investigate the synergistic impact of microalgae biomass during its co-pyrolysis with swine manure digestate. TG and DTG profiles of plain and blended samples showed three zones of devolatilization. The volatiles and extractives in the biomass samples enhanced the kinetics of thermal decomposition of biomass blends. The mineral content of the ash in the blends enhanced their kinetics, which is evident based on gas yields and low activation energy with blends when compared to SWD biomass. If heat flow and disorder change are comprehensively evaluated, the higher activation ∆*G* values indicated favorability for the reactions to happen. The synergy between biomasses was evident in the gas evolution trends, as the total gas yield was noticed to increase with increase in the proportion of MA in the blends. In addition, the second evolution peaks of CO_2_ and CO were found to decrease with a rise in the proportion of MA.

## Data Availability

Not applicable.
